# Transdifferentiation of differentiated stem cells contributes to remyelination

**DOI:** 10.1186/s13287-015-0186-y

**Published:** 2015-10-05

**Authors:** Bharath Chelluboina, Dzung H. Dinh, Krishna Kumar Veeravalli

**Affiliations:** Department of Cancer Biology and Pharmacology, University of Illinois College of Medicine at Peoria, 1 Illini Drive, Peoria, IL 61605 USA; Department of Neurosurgery, University of Illinois College of Medicine at Peoria, 530 N.E. Glen Oak Ave., Peoria, IL 61637 USA; Illinois Neurological Institute, OSF Saint Francis Medical Center, 530 N.E. Glen Oak Ave., Peoria, IL 61637 USA

## Abstract

Evidence suggests that transdifferentiation of mesenchymal stem cells (MSCs) into various neuronal cells contributes to functional recovery after experimental spinal cord injury. Qiu et al. have recently published an exciting article in *Stem Cell Research & Therapy* demonstrating the transdifferentiation of already differentiated MSCs that contributes to remyelination of injured/regenerating axons, and thereby to functional recovery of spinal cord injured animals. The authors highlight the importance of interaction between neurotrophin-3 and tropomyosin receptor kinase C for the observed effects. This study provided important evidence that manipulation of rat bone marrow-derived MSCs before transplantation could enhance the therapeutic benefit of cell-based treatment.

## Introduction

Despite exciting progress in the field of regeneration, functional remyelination has not been demonstrated in the adult injured central nervous system. For transplanted mesenchymal stem cells (MSCs) to effectively reverse traumatic spinal cord injury (SCI), multiple barriers have to be overcome such as low survival of transplanted MSCs, limited neuronal transdifferentiation, remyelination, synaptogenesis, and, not least of all, gliosis. Regardless of the source of MSCs (adipose, bone marrow, or umbilical cord blood), cell-based therapy appeared to be promising in mitigating the extent of SCI, promoting neuronal repair and regeneration. Even though our laboratory has demonstrated the ability of MSCs to transdifferentiate and remyelinate injured axons, the mechanism behind these beneficial effects remains unclear [1–3].

## Main text

Several recent studies have shown that manipulated Schwann cells (SCs) and MSCs which overexpressed neurotrophin-3 (NT-3) and tropomyosin receptor kinase C (TrkC), alone or in concert in co-cultured milieu, promoted survivability, neuronal transdifferentiation, remyelination, and synaptogenesis and attenuated the gliosis process [4–8]. This study by Qiu et al. [9] used only MSCs, instead of mixed SCs and MSCs as used in other studies. The rat mesenchymal stem cells (rMSCs) were genetically manipulated to overexpress either NT-3 or its high-affinity receptor TrkC and allowed the cells to differentiate in a three-dimensional gelatin sponge scaffold. Fourteen days after culture in the scaffold, none of the rMSCs was positive for astrocyte and oligodendrocyte markers. More than 70 % of either manipulated or nonmanipulated rMSCs expressed immature neuronal markers. However, only 7 % of the nonmanipulated rMSCs transdifferentiated into mature neuronal marker, as compared with 44 % and 45 % in NT-3-overexpressed rMSCs and TrkC-overexpressed rMSCs, respectively. The percentage of rMSCs positive for mature neuronal marker increased to 68 % (*p* < 0.05 vs. other groups) when NT-3-overexpressing and TrkC-overexpressing rMSCs were cultured together. This study highlighted the importance of NT-3/TrkC interaction in improving the transdifferentiating potential of rMSCs to neural-like cells. Although these results are interesting, we still do not understand why the nonmanipulated rMSCs were positive only for immature neuronal marker and not for astrocyte or oligodendrocyte markers.

NT-3 is a member of one of the four known neurotrophins, which include nerve growth factor, brain-derived neurotrophic factor, and neurotrophin 4/5. NT-3 acts very strongly on neurons within the corticospinal tract [10]. Binding of NT-3 to its high-affinity receptor TrkC has been shown to trigger axonal growth, maturation, and plasticity of synapses [11]. Binding of NT-3 to TrkC also induces phosphorylation of intracellular tyrosine residues that block caspase activation associated with cell death [12]. Qiu et al. have provided evidence that NT-3/TrkC binding caused the transdifferentiation of already differentiated rMSCs in the injured rat spinal cord. The most interesting outcome of this study is the transdifferentiation of rMSC-derived neural-like cells into myelin-forming cells after their transplantation in the injured rat spinal cord. Here, the authors highlighted the importance and contribution of NT-3/TrkC interaction to the loss of neural phenotypes in rMSC-derived neural-like cells, the transdifferentiation of the differentiated rMSCs into myelin-forming cells, the formation of myelin sheaths around the injured/regenerating axons, and the locomotor recovery of spinal cord injured rats. However, the authors did not elucidate the underlying mechanism of transdifferentiation of already differentiated cells. It could be speculated that the local microenvironment of the demyelinated region may be more conducive to induce the transdifferentiation of rMSC-derived neural-like cells into oligodendrocytes. Another study published by the same group of researchers demonstrated that the addition of exogenous NT-3 into the cultured TrkC-overexpressing rMSCs promoted their transdifferentiation into oligodendrocytes [7]. Currently, we do not know why NT-3/TrkC binding in vitro led to transdifferentiation of TrkC-overexpressing rMSCs into myelin-forming cells and the associated mechanisms. It is possible that binding of NT-3/TrkC activates downstream signaling of the mitogen-activated protein kinase kinase (MEK) –extracellular signal-regulated kinase (ERK) pathway [13].

## Conclusions

Further studies are warranted to better understand the underlying molecular mechanisms of NT-3/TrkC signaling that leads to transdifferentiation of MSCs.

## Abbreviations

MSC: Mesenchymal stem cell
NT-3: Neurotrophin-3
rMSC: Rat mesenchymal stem cell
SC: Schwann cell
SCI: Spinal cord injury
TrkC: Tropomyosin receptor kinase C
